# Supplementary material for: Lipotoxic Impairment of Mitochondrial Function in β-Cells: A Review
Source: Antioxidants (Basel). 2021 Feb 15;10(2):293. doi: 10.3390/antiox10020293 (PMC7919463; doi:10.3390/antiox10020293)
Supplement: Supplementary file 1 [file antioxidants-10-00293-s001.pdf]

Supplementary material

Lipotoxic Impairment of Mitochondrial Function in β-cells: a Review

Axel Römer, Thomas Linn and Sebastian F. Petry

Supplement S1: Articles included after literature search on PubMed (n = 130)

Assali, E.A.; Shlomo, D.; Zeng, J.; Taddeo, E.P.; Trudeau, K.M.; Erion, K.A.; Colby, A.H.; Grinstaff, M.W.; Liesa, M.; Las, G.; et al. Nanoparticle-mediated lysosomal reacidification restores mitochondrial turnover and function in β cells under lipotoxicity. *FASEB J.* **2019**, *33*, 4154–4165, doi:10.1096/fj.201801292R.

Barbera, M.; Fierabracci, V.; Novelli, M.; Bombara, M.; Masiello, P.; Bergamini, E.; De Tata, V. Dexamethasone-induced insulin resistance and pancreatic adaptive response in aging rats are not modified by oral vanadyl sulfate treatment. *Eur. J. Endocrinol.* **2001**, *145*, 799–806, doi:10.1530/eje.0.1450799.

Barlow, J.; Affourtit, C. Novel insights into pancreatic β-cell glucolipotoxicity from real-time functional analysis of mitochondrial energy metabolism in INS-1E insulinoma cells. *Biochem. J.* **2013**, *456*, 417–426, doi:10.1042/BJ20131002.

Boucher, A.; Lu, D.; Burgess, S.C.; Telemaque-Potts, S.; Jensen, M. V.; Mulder, H.; Wang, M.Y.; Unger, R.H.; Sherry, A.D.; Newgard, C.B. Biochemical mechanism of lipid-induced impairment of glucose-stimulated insulin secretion and reversal with a malate analogue. *J. Biol. Chem.* **2004**, *279*, 27263–27271, doi:10.1074/jbc.M401167200.

Briaud, I.; Harmon, J.S.; Kelpe, C.L.; Segu, V.B.G.; Poitout, V. Lipotoxicity of the pancreatic β-cell is associated with glucose-dependent esterification of fatty acids into neutral lipids. *Diabetes* **2001**, *50*, 315–321, doi:10.2337/diabetes.50.2.315.

Carlessi, R.; Rowlands, J.; Ellison, G.; Helena de Oliveira Alves, H.; Newsholme, P.; Mamotte, C. Glutamine deprivation induces metabolic adaptations associated with beta cell dysfunction and exacerbate lipotoxicity. *Mol. Cell. Endocrinol.* **2019**, *491*, 110433, doi:10.1016/j.mce.2019.04.013.

Chen, J.; Fontes, G.; Saxena, G.; Poitout, V.; Shalev, A. Lack of TXNIP protects against mitochondria-mediated apoptosis but not against fatty acid-induced ER stress-mediated β-cell death. *Diabetes* **2010**, *59*, 440–447, doi:10.2337/db09-0949.

Chen, Y.; Ren, Q.; Zhou, Z.; Deng, L.; Hu, L.; Zhang, L.; Li, Z. HWL-088, a new potent free fatty acid receptor 1 (FFAR1) agonist, improves glucolipid metabolism and acts additively with metformin in ob/ob diabetic mice. *Br. J. Pharmacol.* **2020**, *177*, 2286–2302, doi:10.1111/bph.14980.

Cheon, H.G.; Cho, Y.S. Protection of palmitic acid-mediated lipotoxicity by arachidonic acid via channeling of palmitic acid into triglycerides in C2C12. *J. Biomed. Sci.* **2014**, *21*, doi:10.1186/1423-0127-21-13.

Cho, Y.S.; Kim, C.H.; Kim, K.Y.; Cheon, H.G. Protective effects of arachidonic acid against palmitic acid-mediated lipotoxicity in HIT-T15 cells. *Mol. Cell. Biochem.* **2012**, *364*, 19–28, doi:10.1007/s11010-011-1200-z.

Ciregia, F.; Giusti, L.; Ronci, M.; Bugliani, M.; Piga, I.; Pieroni, L.; Rossi, C.; Marchetti, P.; Urbani, A.; Lucacchini, A. Glucagon-like peptide 1 protects INS-1E mitochondria against palmitate-mediated beta-cell dysfunction: A proteomic study. *Mol. Biosyst.* **2015**, *11*, 1696–1707, doi:10.1039/c5mb00022j.

Ciregia, F.; Bugliani, M.; Ronci, M.; Giusti, L.; Boldrini, C.; Mazzoni, M.R.; Mossuto, S.; Grano, F.; Cnop, M.; Marselli, L.; et al. Palmitate-induced lipotoxicity alters acetylation of multiple proteins in clonal β cells and human pancreatic islets. *Sci. Rep.* **2017**, *7*, 13445, doi:10.1038/s41598-017-13908-w.

Cnop, M.; Hannaert, J.C.; Hoorens, A.; Eizirik, D.L.; Pipeleers, D.G. Inverse Relationship between Cytotoxicity of Free Fatty Acids in Pancreatic Islet Cells and Cellular Triglyceride Accumulation. *Diabetes* **2001**, *50*, 1771–1777, doi:10.2337/diabetes.50.8.1771.

Cnop, M.; Welsh, N.; Jonas, J.C.; Jörns, A.; Lenzen, S.; Eizirik, D.L. Mechanisms of pancreatic β-cell death in type 1 and type 2 diabetes: Many differences, few similarities. *Diabetes* **2005**, *54 Suppl*, 97–107, doi:10.2337/diabetes.54.suppl_2.S97.

Cripps, M.J.; Bagnati, M.; Jones, T.A.; Ogunkolade, B.W.; Sayers, S.R.; Caton, P.W.; Hanna, K.; Billacura, M.P.; Fair, K.; Nelson, C.; et al. Identification of a subset of trace amine-associated receptors and ligands as

potential modulators of insulin secretion. *Biochem. Pharmacol.* **2020**, *171*, 113685, doi:10.1016/j.bcp.2019.113685.

Cunha, D.A.; Ladrière, L.; Ortis, F.; Igoillo-Esteve, M.; Gurzov, E.N.; Lupi, R.; Marchetti, P.; Eizirik, D.L.; Cnop, M. Glucagon-like peptide-1 agonists protect pancreatic β-cells from lipotoxic endoplasmic reticulum stress through upregulation of BIP and JunB. *Diabetes* **2009**, *58*, 2851–2862, doi:10.2337/db09-0685.

Cunha, D.A.; Igoillo-Esteve, M.; Gurzov, E.N.; Germano, C.M.; Naamane, N.; Marhfour, I.; Fukaya, M.; Vanderwinden, J.M.; Gysemans, C.; Mathieu, C.; et al. Death protein 5 and p53-upregulated modulator of apoptosis mediate the endoplasmic reticulum stress-mitochondrial dialog triggering lipotoxic rodent and human β-cell apoptosis. *Diabetes* **2012**, *61*, 2763–2775, doi:10.2337/db12-0123.

Cunha, D.A.; Cito, M.; Grieco, F.A.; Cosentino, C.; Danilova, T.; Ladrière, L.; Lindahl, M.; Domanskyi, A.; Bugliani, M.; Marchetti, P.; et al. Pancreatic β-cell protection from inflammatory stress by the endoplasmic reticulum proteins thrombospondin 1 and mesencephalic astrocyte-derived neutrotrophic factor (MANF). *J. Biol. Chem.* **2017**, *292*, 14977–14988, doi:10.1074/jbc.M116.769877.

El-Assaad, W.; Buteau, J.; Peyot, M.L.; Nolan, C.; Roduit, R.; Hardy, S.; Joly, E.; Dbaibo, G.; Rosenberg, L.; Prentki, M. Saturated fatty acids synergize with elevated glucose to cause pancreatic β-cell death. *Endocrinology* **2003**, *144*, 4154–4163, doi:10.1210/en.2003-0410.

Elsner, M.; Gehrmann, W.; Lenzen, S. Peroxisome-generated hydrogen peroxide as important mediator of lipotoxicity in insulin-producing cells. *Diabetes* **2011**, *60*, 200–208, doi:10.2337/db09-1401.

Frigerio, F.; Chaffard, G.; Berwaer, M.; Maechler, P. The antiepileptic drug topiramate preserves metabolism-secretion coupling in insulin secreting cells chronically exposed to the fatty acid oleate. *Biochem. Pharmacol.* **2006**, *72*, 965–973, doi:10.1016/j.bcp.2006.07.013.

Fujitani, Y.; Ebato, C.; Uchida, T.; Kawamori, R.; Watada, H. β-cell autophagy: A novel mechanism regulating β-cell function and mass- Lessons from β-cell-specific Atg7-deficient mice. *Islets* **2009**, *1*, 151–153, doi:10.4161/isl.1.2.9057.

Gehrmann, W.; Elsner, M.; Lenzen, S. Role of metabolically generated reactive oxygen species for lipotoxicity in pancreatic β-cells. *Diabetes. Obes. Metab.* **2010**, *12*, 149–158, doi:10.1111/j.1463-1326.2010.01265.x.

Gharib, E.; Kouhsari, S.M. Study of the antidiabetic activity of Punica granatum L. Fruits aqueous extract on the alloxan-diabetic wistar rats. *Iran. J. Pharm. Res.* **2019**, *18*, 358–368, doi:10.22037/ijpr.2019.2324.

Gharib, E.; Kouhsari, S.M.; Izad, M. Punica granatum L. Fruit aqueous extract suppresses reactive oxygen species-mediated p53/p65/miR-145 expressions followed by Elevated Levels of irs-1 in alloxan-diabetic rats. *Cell J.* **2018**, *19*, 520–527, doi:10.22074/cellj.2018.4550.

Green, C.D.; Jump, D.B.; Olson, L.K. Elevated insulin secretion from liver X receptor-activated pancreatic β-cells involves increased de novo lipid synthesis and triacylglyceride turnover. *Endocrinology* **2009**, *150*, 2637–2645, doi:10.1210/en.2008-1039.

Grishko, V.; Rachek, L.; Musiyenko, S.; LeDoux, S.P.; Wilson, G.L. Involvement of mtDNA damage in free fatty acid-induced apoptosis. *Free Radic. Biol. Med.* **2005**, *38*, 755–762, doi:10.1016/j.freeradbiomed.2004.11.023.

Grubelnik, V.; Markovič, R.; Lipovšek, S.; Leitinger, G.; Gosak, M.; Dolenšek, J.; Valladolid-Acebes, I.; Berggren, P.O.; Stožer, A.; Perc, M.; et al. Modelling of dysregulated glucagon secretion in type 2 diabetes by considering mitochondrial alterations in pancreatic α-cells. *R. Soc. Open Sci.* **2020**, *7*, 191171, doi:10.1098/rsos.191171.

Grubelnik, V.; Zmazek, J.; Markovič, R.; Gosak, M.; Marhl, M. Modelling of energy-driven switch for glucagon and insulin secretion. *J. Theor. Biol.* **2020**, *493*, 110213, doi:10.1016/j.jtbi.2020.110213.

Gu, J.; Wei, Q.; Zheng, H.; Meng, X.; Zhang, J.; Wang, D. Exendin-4 Promotes Survival of Mouse Pancreatic β - Cell Line in Lipotoxic Conditions, through the Extracellular Signal-Related Kinase 1/2 Pathway. *J. Diabetes Res.* **2016**, *2016*, 1–8, doi:10.1155/2016/5294025.

Guo, T.; Liu, T.; Sun, Y.; Liu, X.; Xiong, R.; Li, H.; Li, Z.; Zhang, Z.; Tian, Z.; Tian, Y. Sonodynamic therapy inhibits palmitate-induced beta cell dysfunction via PINK1/Parkin-dependent mitophagy. *Cell Death Dis.* **2019**, *10*, 457, doi:10.1038/s41419-019-1695-x.

Hansen, J.B.; Dos Santos, L.R.B.; Liu, Y.; Prentice, K.J.; Teudt, F.; Tonnesen, M.; Jonas, J.C.; Wheeler, M.B.; Mandrup-Poulsen, T. Glucolipotoxic conditions induce β-cell iron import, cytosolic ROS formation and apoptosis. *J. Mol. Endocrinol.* **2018**, *61*, 69–77, doi:10.1530/JME-17-0262.

Hao, F.; Kang, J.; Cao, Y.; Fan, S.; Yang, H.; An, Y.; Pan, Y.; Tie, L.; Li, X. Curcumin attenuates palmitate-induced apoptosis in MIN6 pancreatic β-cells through PI3K/Akt/FoxO1 and mitochondrial survival pathways. *Apoptosis* **2015**, *20*, 1420–1432, doi:10.1007/s10495-015-1150-0.

Hennige, A.M.; Ranta, F.; Heinzelmann, I.; Düfer, M.; Michael, D.; Braumüller, H.; Lutz, S.Z.; Lammers, R.; Drews, G.; Bosch, F.; et al. Overexpression of kinase-negative protein kinase Cδ in pancreatic β-cells protects mice from diet-induced glucose intolerance and β-cell dysfunction. *Diabetes* **2010**, *59*, 119–127, doi:10.2337/db09-0512.

Higa, M.; Zhou, Y.T.; Ravazzola, M.; Baetens, D.; Orci, L.; Unger, R.H. Troglitazone prevents mitochondrial alterations, β cell destruction, and diabetes in obese prediabetic rats. *Proc. Natl. Acad. Sci. U. S. A.* **1999**, *96*, 11513–11518, doi:10.1073/pnas.96.20.11513.

Hirota, N.; Otabe, S.; Nakayama, H.; Yuan, X.; Yamada, K. Sequential activation of caspases and synergistic β-cell cytotoxicity by palmitate and anti-Fas antibodies. *Life Sci.* **2006**, *79*, 1312–1316, doi:10.1016/j.lfs.2006.03.048.

Hoa Nguyen, K.; Yao, X.H.; Erickson, A.G.; Mishra, S.; Grégoire Nyomba, B.L. Glucose intolerance in aging male igfbp-3 transgenic mice: Differential effects of human igfbp-3 and its mutant igfbp-3 devoid of igf binding ability. *Endocrinology* **2015**, *156*, 462–474, doi:10.1210/en.2014-1271.

Hu, H.Q.; Qiao, J.T.; Liu, F.Q.; Wang, J.B.; Sha, S.; He, Q.; Cui, C.; Song, J.; Zang, N.; Wang, L.S.; et al. The STING-IRF3 pathway is involved in lipotoxic injury of pancreatic β cells in type 2 diabetes. *Mol. Cell. Endocrinol.* **2020**, 110890, doi:10.1016/j.mce.2020.110890.

Hu, M.; Lin, H.; Yang, L.; Cheng, Y.; Zhang, H. Interleukin-22 restored mitochondrial damage and impaired glucose-stimulated insulin secretion through down-regulation of uncoupling protein-2 in INS-1 cells. *J. Biochem.* **2017**, *161*, 433–439, doi:10.1093/jb/mvw084.

Huang, C.N.; Wang, C.J.; Lee, Y.J.; Peng, C.H. Active subfractions of Abelmoschus esculentus substantially prevent free fatty acid-induced ß cell apoptosis via inhibiting dipeptidyl peptidase-4. *PLoS One* **2017**, *12*, e0180285, doi:10.1371/journal.pone.0180285.

Ježek, J.; Dlasková, A.; Zelenka, J.; Jabůrek, M.; Ježek, P. H2O2-activated mitochondrial phospholipase iPLA2γ prevents lipotoxic oxidative stress in synergy with UCP2, amplifies signaling via G-protein-coupled receptor GPR40, and regulates insulin secretion in pancreatic β-cells. *Antioxidants Redox Signal.* **2015**, *23*, 958–972, doi:10.1089/ars.2014.6195.

Ježek, P.; Jabůrek, M.; Holendová, B.; Plecitá-Hlavatá, L. Fatty Acid-Stimulated Insulin Secretion vs. Lipotoxicity. *Molecules* **2018**, *23*, 1483, doi:10.3390/molecules23061483.

Jiang, L.; Wan, J.; Ke, L.Q.; Lü, Q.G.; Tong, N.W. Activation of PPARδ promotes mitochondrial energy metabolism and decreases basal insulin secretion in palmitate-treated β-cells. *Mol. Cell. Biochem.* **2010**, *343*, 249–256, doi:10.1007/s11010-010-0520-8.

Joseph, J.W.; Koshkin, V.; Saleh, M.C.; Sivitz, W.I.; Zhang, C.Y.; Lowell, B.B.; Chan, C.B.; Wheeler, M.B. Free fatty acid-induced β-cell defects are dependent on uncoupling protein 2 expression. *J. Biol. Chem.* **2004**, *279*, 51049–51056, doi:10.1074/jbc.M409189200.

Jung, I.R.; Choi, S.E.; Jung, J.G.; Lee, S.A.; Han, S.J.; Kim, H.J.; Kim, D.J.; Lee, K.W.; Kang, Y. Involvement of iron depletion in palmitate-induced lipotoxicity of beta cells. *Mol. Cell. Endocrinol.* **2015**, *407*, 74–84, doi:10.1016/j.mce.2015.03.007.

Kato, T.; Shimano, H.; Yamamoto, T.; Ishikawa, M.; Kumadaki, S.; Matsuzaka, T.; Nakagawa, Y.; Yahagi, N.; Nakakuki, M.; Hasty, A.H.; et al. Palmitate impairs and eicosapentaenoate restores insulin secretion through regulation of SREBP-1c in pancreatic islets. *Diabetes* **2008**, *57*, 2382–2392, doi:10.2337/db06-1806.

Kim, M.; Lee, J.S.; Oh, J.E.; Nan, J.; Lee, H.; Jung, H.S.; Chung, S.S.; Park, K.S. SIRT3 overexpression attenuates palmitate-induced pancreatic β-cell dysfunction. *PLoS One* **2015**, *10*, e0124744, doi:10.1371/journal.pone.0124744.

Köhnke, R.; Mei, J.; Park, M.J.; York, D.A.; Erlanson-Albertsson, C. Fatty acids and glucose in high concentration down-regulates ATP synthase β-subunit protein expression in INS-1 cells. *Nutr. Neurosci.* **2007**, *10*, 273–278, doi:10.1080/10284150701745910.

Komatsu, M.; Yajima, H.; Yamada, S.; Kaneko, T.; Sato, Y.; Yamauchi, K.; Hashizume, K.; Aizawa, T. Augmentation of Ca2+-stimulated insulin release by glucose and long- chain fatty acids in rat pancreatic islets: Free fatty acids mimic ATP- sensitive K+ channel-independent insulinotropic action of glucose. *Diabetes* **1999**, *48*, 1543–1549, doi:10.2337/diabetes.48.8.1543.

Koshkin, V.; Wang, X.; Scherer, P.E.; Chan, C.B.; Wheeler, M.B. Mitochondrial functional state in clonal pancreatic β-cells exposed to free fatty acids. *J. Biol. Chem.* **2003**, *278*, 19709–19715, doi:10.1074/jbc.M209709200.

Koshkin, V.; Dai, F.F.; Robson-Doucette, C.A.; Chan, C.B.; Wheeler, M.B. Limited mitochondrial permeabilization is an early manifestation of palmitate-induced lipotoxicity in pancreatic β-cells. *J. Biol. Chem.* **2008**, *283*, 7936–7948, doi:10.1074/jbc.M705652200.

Koulajian, K.; Ivovic, A.; Ye, K.; Desai, T.; Shah, A.; George Fantus, I.; Ran, Q.; Giacca, A. Overexpression of glutathione peroxidase 4 prevents β-cell dysfunction induced by prolonged elevation of lipids in vivo. *Am. J. Physiol. - Endocrinol. Metab.* **2013**, *305*, E254-62, doi:10.1152/ajpendo.00481.2012.

Kristinsson, H.; Sargsyan, E.; Manell, H.; Smith, D.M.; Göpel, S.O.; Bergsten, P. Basal hypersecretion of glucagon and insulin from palmitate-exposed human islets depends on FFAR1 but not decreased somatostatin secretion. *Sci. Rep.* **2017**, *7*, 4657, doi:10.1038/s41598-017-04730-5.

Kwak, H.J.; Yang, D.; Hwang, Y.; Jun, H.S.; Cheon, H.G. Baicalein protects rat insulinoma INS-1 cells from palmitate-induced lipotoxicity by inducing HO-1. *PLoS One* **2017**, *12*, e0176432, doi:10.1371/journal.pone.0176432.

Ladrière, L.; Igoillo-Esteve, M.; Cunha, D.A.; Brion, J.P.; Bugliani, M.; Marchetti, P.; Eizirik, D.L.; Cnop, M. Enhanced signaling downstream of ribonucleic acid-activated protein kinase-like endoplasmic reticulum kinase potentiates lipotoxic endoplasmic reticulum stress in human islets. *J. Clin. Endocrinol. Metab.* **2010**, *95*, 1442–1449, doi:10.1210/jc.2009-2322.

Lameloise, N.; Muzzin, P.; Prentki, M.; Assimacopoulos-Jeannet, F. Uncoupling protein 2: A possible link between fatty acid excess and impaired glucose-induced insulin secretion? *Diabetes* **2001**, *50*, 803–809, doi:10.2337/diabetes.50.4.803.

Laporte, A.; Lortz, S.; Schaal, C.; Lenzen, S.; Elsner, M. Hydrogen peroxide permeability of cellular membranes in insulin-producing cells. *Biochim. Biophys. Acta - Biomembr.* **2020**, *1862*, 183096, doi:10.1016/j.bbamem.2019.183096.

Lee, J.H.; Jung, I.R.; Choi, S.E.; Lee, S.M.; Lee, S.J.; Han, S.J.; Kim, H.J.; Kim, D.J.; Lee, K.W.; Kang, Y. Toxicity generated through inhibition of pyruvate carboxylase and carnitine palmitoyl transferase-1 is similar to high glucose/palmitate-induced glucolipotoxicity in INS-1 beta cells. *Mol. Cell. Endocrinol.* **2014**, *383*, 48–59, doi:10.1016/j.mce.2013.12.002.

Lee, K.M.; Seo, Y.J.; Kim, M.K.; Seo, H.A.; Jeong, J.Y.; Choi, H.S.; Lee, I.K.; Park, K. gyu Mediation of glucolipotoxicity in INS-1 rat insulinoma cells by small heterodimer partner interacting leucine zipper protein (SMILE). *Biochem. Biophys. Res. Commun.* **2012**, *419*, 768–773, doi:10.1016/j.bbrc.2012.02.098.

Li, F.; Munsey, T.S.; Sivaprasadarao, A. TRPM2-mediated rise in mitochondrial Zn 2+ promotes palmitate-induced mitochondrial fission and pancreatic β-cell death in rodents. *Cell Death Differ.* **2017**, *24*, 1999–2012, doi:10.1038/cdd.2017.118.

Li, Z.; Zhou, Z.; Huang, G.; Hu, F.; Xiang, Y.; He, L. Exendin-4 Protects Mitochondria from Reactive Oxygen Species Induced Apoptosis in Pancreatic Beta Cells. *PLoS One* **2013**, *8*, e76172, doi:10.1371/journal.pone.0076172.

Li, Z.; Liu, C.; Zhou, Z.; Hu, L.; Deng, L.; Ren, Q.; Qian, H. A novel FFA1 agonist, CPU025, improves glucose-lipid metabolism and alleviates fatty liver in obese-diabetic (ob/ob) mice. *Pharmacol. Res.* **2020**, *153*, 104679, doi:10.1016/j.phrs.2020.104679.

Li, Z.; Zhou, Z.; Hu, L.; Deng, L.; Ren, Q.; Zhang, L. ZLY032, the first-in-class dual FFA1/PPARδ agonist, improves glucolipid metabolism and alleviates hepatic fibrosis. *Pharmacol. Res.* **2020**, *159*, 105035, doi:10.1016/j.phrs.2020.105035.

Liu, C.G.; Ma, Y.P.; Zhang, X.J. Effects of mulberry leaf polysaccharide on oxidative stress in pancreatic β-cells of type 2 diabetic rats. *Eur. Rev. Med. Pharmacol. Sci.* **2017**, *21*, 2482–2488.

Liu, L.; Liang, C.; Mei, P.; Zhu, H.; Hou, M.; Yu, C.; Song, Z.; Bao, Y.; Huang, Y.; Yi, J.; et al. Dracorhodin perchlorate protects pancreatic β-cells against glucotoxicity- or lipotoxicity-induced dysfunction and apoptosis in vitro and in vivo. *FEBS J.* **2019**, *286*, 3718–3736, doi:10.1111/febs.15020.

Ly, L.D.; Ly, D. Da; Nguyen, N.T.; Kim, J.H.; Yoo, H.; Chung, J.; Lee, M.S.; Cha, S.K.; Park, K.S. Mitochondrial Ca2+ Uptake Relieves Palmitate-Induced Cytosolic Ca2+ Overload in MIN6 Cells. *Mol. Cells* **2020**, *43*, 66–75, doi:10.14348/molcells.2019.0223.

Maassen, J.A.; 'T Hart, L.M.; Janssen, G.M.C.; Reiling, E.; Romijn, J.A.; Lemkes, H.H. Mitochondrial diabetes and its lessons for common Type 2 diabetes. *Biochem. Soc. Trans.* **2006**, *34*, 819–823, doi:10.1042/BST0340819.

Maczewsky, J.; Sikimic, J.; Bauer, C.; Krippeit-Drews, P.; Wolke, C.; Lendeckel, U.; Barthlen, W.; Drews, G. The LXR ligand T0901317 acutely inhibits insulin secretion by affecting mitochondrial metabolism. *Endocrinology* **2017**, *158*, 2145–2154, doi:10.1210/en.2016-1941.

Maedler, K.; Spinas, G.A.; Dyntar, D.; Moritz, W.; Kaiser, N.; Donath, M.Y. Distinct effects of saturated and monounsaturated fatty acids on β-cell turnover and function. *Diabetes* **2001**, *50*, 69–76, doi:10.2337/diabetes.50.1.69.

Maedler, K.; Oberholzer, J.; Bucher, P.; Spinas, G.A.; Donath, M.Y. Monounsaturated fatty acids prevent the deleterious effects of palmitate and high glucose on human pancreatic β-cell turnover and function. *Diabetes* **2003**, *52*, 726–733, doi:10.2337/diabetes.52.3.726.

Maestre, I.; Jordán, J.; Calvo, S.; Reig, J.A.; Ceña, V.; Soria, B.; Prentki, M.; Roche, E. Mitochondrial dysfunction is involved in apoptosis induced by serum withdrawal and fatty acids in the β-cell line INS-1. *Endocrinology* **2003**, *144*, 335–345, doi:10.1210/en.2001-211282.

Maris, M.; Robert, S.; Waelkens, E.; Derua, R.; Hernangomez, M.H.; D'Hertog, W.; Cnop, M.; Mathieu, C.; Overbergh, L. Role of the saturated nonesterified fatty acid palmitate in beta cell dysfunction. *J. Proteome Res.* **2013**, *12*, 347–362, doi:10.1021/pr300596g.

McCarty, M.F. A chlorogenic acid-induced increase in GLP-1 production may mediate the impact of heavy coffee consumption on diabetes risk. *Med. Hypotheses* **2005**, *64*, 848–853, doi:10.1016/j.mehy.2004.03.037.

Medvedev, A. V.; Robidoux, J.; Bai, X.; Cao, W.; Floering, L.M.; Daniel, K.W.; Collins, S. Regulation of the uncoupling protein-2 gene in INS-1 β-cells by oleic acid. *J. Biol. Chem.* **2002**, *277*, 42639–42644, doi:10.1074/jbc.M208645200.

Mir, S.U.R.; George, N.M.; Zahoor, L.; Harms, R.; Guinn, Z.; Sarvetnick, N.E. Inhibition of autophagic turnover in β-cells by fatty acids and glucose leads to apoptotic cell death. *J. Biol. Chem.* **2015**, *290*, 6071–6085, doi:10.1074/jbc.M114.605345.

Molina, A.J.A.; Wikstrom, J.D.; Stiles, L.; Las, G.; Mohamed, H.; Elorza, A.; Walzer, G.; Twig, G.; Katz, S.; Corkey, B.E.; et al. Mitochondrial networking protects β-cells from nutrient-induced apoptosis. *Diabetes* **2009**, *58*, 2303–2315, doi:10.2337/db07-1781.

Muzumdar, R.; Ma, X.; Atzmon, G.; Vuguin, P.; Yang, X.; Barzilai, N. Decrease in Glucose-Stimulated Insulin Secretion with Aging Is Independent of Insulin Action. *Diabetes* **2004**, *53*, 441–446, doi:10.2337/diabetes.53.2.441.

Nagaraju, R.; Rajini, P.S. Adaptive response of rat pancreatic β-cells to insulin resistance induced by monocrotophos: Biochemical evidence. *Pestic. Biochem. Physiol.* **2016**, *134*, 39–48, doi:10.1016/j.pestbp.2016.04.009.

Nakata, M.; Shintani, N.; Hashimoto, H.; Baba, A.; Yada, T. Intra-islet PACAP protects pancreatic β-cells against glucotoxicity and lipotoxicity. *J. Mol. Neurosci.* **2010**, *42*, 404–410, doi:10.1007/s12031-010-9383-4.

Nakhooda, A.F.; Like, A.A.; Chappel, C.I.; Murray, F.T.; Marliss, E.B. The spontaneously diabetic Wistar rat. Metabolic and morphologic studies. *Diabetes* **1977**, *26*, 100–112, doi:10.2337/diab.26.2.100.

Oberhauser, L.; Granziera, S.; Colom, A.; Goujon, A.; Lavallard, V.; Matile, S.; Roux, A.; Brun, T.; Maechler, P. Palmitate and oleate modify membrane fluidity and kinase activities of INS-1E β-cells alongside altered metabolism-secretion coupling. *Biochim. Biophys. Acta - Mol. Cell Res.* **2020**, *1867*, 118619, doi:10.1016/j.bbamcr.2019.118619.

Oropeza, D.; Jouvet, N.; Bouyakdan, K.; Perron, G.; Ringuette, L.J.; Philipson, L.H.; Kiss, R.S.; Poitout, V.; Alquier, T.; Estall, J.L. PGC-1 coactivators in β-cells regulate lipid metabolism and are essential for insulin secretion coupled to fatty acids. *Mol. Metab.* **2015**, *4*, 811–822, doi:10.1016/j.molmet.2015.08.001.

Paolisso, G.; Giugliano, D.; D'Amore, A.; Varricchio, M.; Galzerano, D.; D'Onofrio, F.; Balbi, V. Daily vitamin E supplements improve metabolic control but not insulin secretion in elderly type II diabetic patients. *Diabetes Care* **1993**, *16*, 1433–1437, doi:10.2337/diacare.16.11.1433.

Peng, L.; Men, X.; Zhang, W.; Wang, H.; Xu, S.; Fang, Q.; Liu, H.; Yang, W.; Lou, J. Involvement of Dynamin-Related Protein 1 in Free Fatty Acid-Induced INS-1-Derived Cell Apoptosis. *PLoS One* **2012**, *7*, e49258, doi:10.1371/journal.pone.0049258.

Petcherski, A.; Trudeau, K.M.; Wolf, D.M.; Segawa, M.; Lee, J.; Taddeo, E.P.; Deeney, J.T.; Liesa, M. Elamipretide Promotes Mitophagosome Formation and Prevents Its Reduction Induced by Nutrient Excess in INS1 β-cells. *J. Mol. Biol.* **2018**, *430*, 4823–4833, doi:10.1016/j.jmb.2018.10.020.

Plaisance, V.; Perret, V.; Favre, D.; Abderrahmani, A.; Yang, J.Y.; Widmann, C.; Regazzi, R. Role of the transcriptional factor C/EBPβ in free fatty acid-elicited β-cell failure. *Mol. Cell. Endocrinol.* **2009**, *305*, 47–55, doi:10.1016/j.mce.2008.12.005.

Plötz, T.; von Hanstein, A.S.; Krümmel, B.; Laporte, A.; Mehmeti, I.; Lenzen, S. Structure-toxicity relationships of saturated and unsaturated free fatty acids for elucidating the lipotoxic effects in human EndoC-βH1 beta-cells. *Biochim. Biophys. Acta - Mol. Basis Dis.* **2019**, *1865*, 165525, doi:10.1016/j.bbadis.2019.08.001.

Poitout, V. β-Cell Lipotoxicity: Burning Fat into Heat? *Endocrinology* **2004**, *145*, 3563–3565, doi:10.1210/en.2004-0479.

Prause, M.; Christensen, D.P.; Billestrup, N.; Mandrup-Poulsen, T. JNK1 protects against glucolipotoxicity-mediated beta-cell apoptosis. *PLoS One* **2014**, *9*, e87067, doi:10.1371/journal.pone.0087067.

Pujol, J.B.; Christinat, N.; Ratinaud, Y.; Savoia, C.; Mitchell, S.E.; Dioum, E.H.M. Coordination of GPR40 and ketogenesis signaling by medium chain fatty acids regulates beta cell function. *Nutrients* **2018**, *10*, 473, doi:10.3390/nu10040473.

Qi, Y.; Chen, J.; Lay, A.; Don, A.; Vadas, M.; Xia, P. Loss of sphingosine kinase 1 predisposes to the onset of diabetes via promoting pancreatic β-cell death in diet-induced obese mice. *FASEB J.* **2013**, *27*, 4294–4304, doi:10.1096/fj.13-230052.

Qureshi, F.M.; Dejene, E.A.; Corbin, K.L.; Nunemaker, C.S. Stress-induced dissociations between intracellular calcium signaling and insulin secretion in pancreatic islets. *Cell Calcium* **2015**, *57*, 366–375, doi:10.1016/j.ceca.2015.03.002.

Rachek, L.I.; Thornley, N.P.; Grishko, V.I.; LeDoux, S.P.; Wilson, G.L. Protection of INS-1 cells from free fatty acid-induced apoptosis by targeting hOGG1 to mitochondria. *Diabetes* **2006**, *55*, 1022–1028, doi:10.2337/diabetes.55.04.06.db05-0865.

Remizov, O.; Jakubov, R.; Düfer, M.; Drews, P.K.; Drews, G.; Waring, M.; Brabant, G.; Wienbergen, A.; Rustenbeck, I.; Schöfl, C. Palmitate-induced Ca2+-signaling in pancreatic beta-cells. *Mol. Cell. Endocrinol.* **2003**, *212*, 1–9, doi:10.1016/j.mce.2003.09.026.

Renganathan, S.; Srivastava, A.; Pillai, R.G. Dhanwantaram kashayam, an Ayurvedic polyherbal formulation, reduces oxidative radicals and reverts lipids profile towards normal in diabetic rats. *Biochem. Biophys. Reports* **2020**, *22*, doi:10.1016/j.bbrep.2020.100755.

Rodríguez-Gutiérrez, R.; Lavalle-González, F.J.; Martínez-Garza, L.E.; Landeros-Olvera, E.; López-Alvarenga, J.C.; Torres-Sepúlveda, M.R.; González-González, J.G.; Mancillas-Adame, L.G.; Salazar-Gonzalez, B.; Villarreal-Pérez, J.Z. Impact of an exercise program on acylcarnitines in obesity: A prospective controlled study. *J. Int. Soc. Sports Nutr.* **2012**, *9*, doi:10.1186/1550-2783-9-22.

Saitoh, Y.; Hongwei, W.; Ueno, H.; Mizuta, M.; Nakazato, M. Candesartan attenuates fatty acid-induced oxidative stress and NAD(P)H oxidase activity in pancreatic β-cells. *Diabetes Res. Clin. Pract.* **2010**, *90*, 54–59, doi:10.1016/j.diabres.2010.06.005.

Saksida, T.; Stosic-Grujicic, S.; Timotijevic, G.; Sandler, S.; Stojanovic, I. Macrophage migration inhibitory factor deficiency protects pancreatic islets from palmitic acid-induced apoptosis. *Immunol. Cell Biol.* **2012**, *90*, 688–698, doi:10.1038/icb.2011.89.

Salgin, B.; Ong, K.K.; Thankamony, A.; Emmett, P.; Wareham, N.J.; Dunger, D.B. Higher fasting plasma free fatty acid levels are associated with lower insulin secretion in children and adults and a higher incidence of type 2 diabetes. *J. Clin. Endocrinol. Metab.* **2012**, *97*, 3302–3309, doi:10.1210/jc.2012-1428.

Santangelo, C.; Matarrese, P.; Masella, R.; Di Carlo, M.C.; Di Lillo, A.; Scazzocchio, B.; Vecci, E.; Malorni, W.; Perfetti, R.; Anastasi, E. Hepatocyte growth factor protects rat RINm5F cell line against free fatty acid-induced apoptosis by counteracting oxidative stress. *J. Mol. Endocrinol.* **2007**, *38*, 147–158, doi:10.1677/jme.1.02133.

Sharma, P.R.; Mackey, A.J.; Dejene, E.A.; Ramadan, J.W.; Langefeld, C.D.; Palmer, N.D.; Taylor, K.D.; Wagenknecht, L.E.; Watanabe, R.M.; Rich, S.S.; et al. An islet-targeted genome-wide association scan identifies novel genes implicated in cytokine-mediated islet stress in type 2 diabetes. *Endocrinology* **2015**, *156*, 3147–3156, doi:10.1210/en.2015-1203.

Song, H.; Wohltmann, M.; Tan, M.; Ladenson, J.H.; Turk, J. Group VIA phospholipase A2 mitigates palmitate-induced β-cell mitochondrial injury and apoptosis. *J. Biol. Chem.* **2014**, *289*, 14194–14210, doi:10.1074/jbc.M114.561910.

Song, Z.; Wang, W.; Li, N.; Yan, S.; Rong, K.; Lan, T.; Xia, P. Sphingosine kinase 2 promotes lipotoxicity in pancreatic β-cells and the progression of diabetes. *FASEB J.* **2019**, *33*, 3636–3646, doi:10.1096/fj.201801496R.

Sun, Y.; Yang, J.; Liu, W.; Yao, G.; Xu, F.; Hayashi, T.; Onodera, S.; Ikejima, T. Attenuating effect of silibinin on palmitic acid-induced apoptosis and mitochondrial dysfunction in pancreatic β-cells is mediated by estrogen receptor alpha. *Mol. Cell. Biochem.* **2019**, *460*, 81–92, doi:10.1007/s11010-019-03572-1.

Syed, I.; Szulc, Z.M.; Ogretmen, B.; Kowluru, A. L-threo-C 6 -pyridinium-ceramide bromide, a novel cationic ceramide, induces NADPH oxidase activation, mitochondrial dysfunction and loss in cell viability in INS 832/13 β-cells. *Cell. Physiol. Biochem.* **2012**, *30*, 1051–1058, doi:10.1159/000341481.

Taddeo, E.P.; Alsabeeh, N.; Baghdasarian, S.; Wikstrom, J.D.; Ritou, E.; Sereda, S.; Erion, K.; Li, J.; Stiles, L.; Abdulla, M.; et al. Mitochondrial proton leak regulated by Cyclophilin D elevates insulin secretion in islets at nonstimulatory glucose levels. *Diabetes* **2020**, *69*, 131–145, doi:10.2337/db19-0379.

Tarasov, A.I.; Semplici, F.; Ravier, M.A.; Bellomo, E.A.; Pullen, T.J.; Gilon, P.; Sekler, I.; Rizzuto, R.; Rutter, G.A. The mitochondrial Ca2+ uniporter MCU is essential for glucose-induced atp increases in pancreatic β-cells. *PLoS One* **2012**, *7*, doi:10.1371/journal.pone.0039722.

Tian, J.Y.; Li, G.; Gu, Y.Y.; Zhang, H.L.; Zhou, W.Z.; Wang, X.; Zhu, H. Da; Luo, T.H.; Luo, M. Role and mechanism of rosiglitazone on the impairment of insulin secretion induced by free fatty acids on isolated rat islets. *Chin. Med. J. (Engl).* **2006**, *119*, 574–580, doi:10.1097/00029330-200604010-00010.

Tordjman, K.; Standley, K.N.; Bernal-Mizrachi, C.; Leone, T.C.; Coleman, T.; Kelly, D.P.; Semenkovich, C.F. PPARα suppresses insulin secretion and induces UCP2 in insulinoma cells. *J. Lipid Res.* **2002**, *43*, 936–943, doi:10.1016/S0022-2275(20)30468-5.

Tran, K.; Li, Y.; Duan, H.; Arora, D.; Lim, H.Y.; Wang, W. Identification of small molecules that protect pancreatic β cells against endoplasmic reticulum stress-induced cell death. *ACS Chem. Biol.* **2014**, *9*, 2796–2806, doi:10.1021/cb500740d.

Tuo, Y.; Wang, D.; Li, S.; Chen, C. Long-term exposure of INS-1 rat insulinoma cells to linoleic acid and glucose in vitro affects cell viability and function through mitochondrial-mediated pathways. *Endocrine* **2011**, *39*, 128–138, doi:10.1007/s12020-010-9432-3.

Um, S.H.; Frigerio, F.; Watanabe, M.; Picard, F.; Joaquin, M.; Sticker, M.; Fumagalli, S.; Allegrini, P.R.; Kozma, S.C.; Auwerx, J.; et al. Absence of S6K1 protects against age- and diet-induced obesity while enhancing insulin sensitivity. *Nature* **2004**, *431*, 200–205, doi:10.1038/nature02866.

Velasquez, C.; Vasquez, J.S.; Balcazar, N. In vitro effect of fatty acids identified in the plasma of obese adolescents on the function of pancreatic β-cells. *Diabetes Metab. J.* **2017**, *41*, 303–315, doi:10.4093/dmj.2017.41.4.303.

Veluthakal, R.; Arora, D.K.; Goalstone, M.L.; Kowluru, R.A.; Kowluru, A. Metabolic Stress Induces Caspase-3 Mediated Degradation and Inactivation of Farnesyl and Geranylgeranyl Transferase Activities in Pancreatic β-Cells. *Cell. Physiol. Biochem.* **2016**, *39*, 2110–2120, doi:10.1159/000447907.

Wan, J.; Jiang, L.; Lü, Q.; Ke, L.; Li, X.; Tong, N. Activation of PPARδ up-regulates fatty acid oxidation and energy uncoupling genes of mitochondria and reduces palmitate-induced apoptosis in pancreatic β-cells. *Biochem. Biophys. Res. Commun.* **2010**, *391*, 1567–1572, doi:10.1016/j.bbrc.2009.12.127.

Wang, W.; Zhang, D.; Zhao, H.; Chen, Y.; Liu, Y.; Cao, C.; Han, L.; Liu, G. Ghrelin inhibits cell apoptosis induced by lipotoxicity in pancreatic β-cell line. *Regul. Pept.* **2010**, *161*, 43–50, doi:10.1016/j.regpep.2009.12.017.

Wang, W.F.; Guo, Y.; Xu, M.; Huang, H.H.; Novikova, L.; Larade, K.; Jiang, Z.G.; Thayer, T.C.; Frontera, J.R.; Aires, D.; et al. Development of diabetes in lean Ncb5or-null mice is associated with manifestations of endoplasmic reticulum and oxidative stress in beta cells. *Biochim. Biophys. Acta - Mol. Basis Dis.* **2011**, *1812*, 1532–1541, doi:10.1016/j.bbadis.2011.07.016.

Wehinger, S.; Ortiz, R.; Díaz, M.I.; Aguirre, A.; Valenzuela, M.; Llanos, P.; Mc Master, C.; Leyton, L.; Quest, A.F.G. Phosphorylation of caveolin-1 on tyrosine-14 induced by ROS enhances palmitate-induced death of beta-pancreatic cells. *Biochim. Biophys. Acta - Mol. Basis Dis.* **2015**, *1852*, 693–708, doi:10.1016/j.bbadis.2014.12.021.

Wei, Q.; Sun, Y.Q.; Zhang, J. Exendin-4, a glucagon-like peptide-1 receptor agonist, inhibits cell apoptosis induced by lipotoxicity in pancreatic β-cell line. *Peptides* **2012**, *37*, 18–24, doi:10.1016/j.peptides.2012.06.018.

Wikstrom, J.D.; Israeli, T.; Bachar-Wikstrom, E.; Swisa, A.; Ariav, Y.; Waiss, M.; Kaganovich, D.; Dor, Y.; Cerasi, E.; Leibowitz, G. AMPK regulates ER morphology and function in stressed pancreatic β-cells via phosphorylation of DRP1. *Mol. Endocrinol.* **2013**, *27*, 1706–1723, doi:10.1210/me.2013-1109.

Winzell, M.S.; Svensson, H.; Enerbäck, S.; Ravnskjaer, K.; Mandrup, S.; Esser, V.; Arner, P.; Alves-Guerra, M.C.; Miroux, B.; Sundler, F.; et al. Pancreatic β-cell lipotoxicity induced by overexpression of hormone-sensitive lipase. *Diabetes* **2003**, *52*, 2057–2065, doi:10.2337/diabetes.52.8.2057.

Yamashita, T.; Eto, K.; Okazaki, Y.; Yamashita, S.; Yamauchi, T.; Sekine, N.; Nagai, R.; Noda, M.; Kadowaki, T. Role of uncoupling protein-2 up-regulation and triglyceride accumulation in impaired glucose-stimulated insulin secretion in a β-cell lipotoxicity model overexpressing sterol regulatory element-binding protein-1c. *Endocrinology* **2004**, *145*, 3566–3577, doi:10.1210/en.2003-1602.

Yuan, H.; Zhang, X.; Huang, X.; Lu, Y.; Tang, W.; Man, Y.; Wang, S.; Xi, J.; Li, J. NADPH oxidase 2-derived reactive oxygen species mediate FFAs-Induced dysfunction and apoptosis of b- Cells via JNK, p38 MAPK and p53 pathways. *PLoS One* **2010**, *5*, e15726, doi:10.1371/journal.pone.0015726.

Zakłos-Szyda, M.; Kowalska-Baron, A.; Pietrzyk, N.; Drzazga, A.; Podsędek, A. Evaluation of viburnum opulus l. Fruit phenolics cytoprotective potential on insulinoma min6 cells relevant for diabetes mellitus and obesity. *Antioxidants* **2020**, *9*, 433, doi:10.3390/antiox9050433.

Zhang, Q.; Cui, Q.; Hou, Y.; Wang, H.; Xu, Y.; Pi, J. The impairment of glucose-stimulated insulin secretion in pancreatic β-cells caused by prolonged glucotoxicity and lipotoxicity is associated with elevated adaptive antioxidant response. *Food Chem. Toxicol.* **2017**, *100*, 161–167, doi:10.1016/j.fct.2016.12.016.

Zhang, Y.; Zhen, W.; Maechler, P.; Liu, D. Small molecule kaempferol modulates PDX-1 protein expression and subsequently promotes pancreatic β-cell survival and function via CREB. *J. Nutr. Biochem.* **2013**, *24*, 638–646, doi:10.1016/j.jnutbio.2012.03.008.

Zhao, Y.; Wang, L.; Qiu, J.; Zha, D.; Sun, Q.; Chen, C. Linoleic Acid Stimulates [Ca2+]i Increase in Rat Pancreatic Beta-Cells through Both Membrane Receptor- and Intracellular Metabolite-Mediated Pathways. *PLoS One* **2013**, *8*, e60255, doi:10.1371/journal.pone.0060255.

Zhou, Y.; Chung, A.C.K.; Fan, R.; Lee, H.M.; Xu, G.; Tomlinson, B.; Chan, J.C.N.; Kong, A.P.S. Sirt3 Deficiency Increased the Vulnerability of Pancreatic Beta Cells to Oxidative Stress-Induced Dysfunction. *Antioxidants Redox Signal.* **2017**, *27*, 962–976, doi:10.1089/ars.2016.6859.

Zhou, Y.P.; Berggren, P.O.; Grill, V. A fatty acid-induced decrease in pyruvate dehydrogenase activity is an important determinant of β-cell dysfunction in the obese diabetic db/db mouse. *Diabetes* **1996**, *45*, 580–586, doi:10.2337/diabetes.45.5.580.

Zhu, Y.; Ren, C.; Zhang, M.; Zhong, Y. Perilipin 5 Reduces Oxidative Damage Associated With Lipotoxicity by Activating the PI3K/ERK-Mediated Nrf2-ARE Signaling Pathway in INS-1 Pancreatic β-Cells. *Front. Endocrinol. (Lausanne).* **2020**, *11*, 166, doi:10.3389/fendo.2020.00166.

© 2021 by the authors. Licensee MDPI, Basel, Switzerland. This article is an open access article distributed under the terms and conditions of the Creative Commons Attribution (CC BY) license (http://creativecommons.org/licenses/by/4.0/).
